# Deep Eutectic Solvents in Solar Energy Technologies

**DOI:** 10.3390/molecules27030709

**Published:** 2022-01-21

**Authors:** Chiara Liliana Boldrini, Andrea Francesca Quivelli, Norberto Manfredi, Vito Capriati, Alessandro Abbotto

**Affiliations:** 1 Solar Energy Research Center MIBSOLAR, Department of Materials Science, INSTM Milano-Bicocca Research Unit, University of Milano-Bicocca, Via Cozzi 55, I-20125 Milano, Italy; chiara.boldrini@unimib.it (C.L.B.); andrea.quivelli@unimib.it (A.F.Q.); norberto.manfredi@unimib.it (N.M.); 2 Dipartimento di Farmacia–Scienze del Farmaco, Università degli Studi di Bari “Aldo Moro”, Via E. Orabona 4, I-70125 Bari, Italy; 3 Consorzio C.I.N.M.P.I.S., Via E. Orabona 4, I-70125 Bari, Italy

**Keywords:** solar energy, deep eutectic solvents, dye-sensitized solar cells, concentrated solar power

## Abstract

Deep Eutectic Solvents (DESs) have been widely used in many fields to exploit their ecofriendly characteristics, from green synthetic procedures to environmentally benign industrial methods. In contrast, their application in emerging solar technologies, where the abundant and clean solar energy is used to properly respond to most important societal needs, is still relatively scarce. This represents a strong limitation since many solar devices make use of polluting or toxic components, thus seriously hampering their eco-friendly nature. Herein, we review the literature, mainly published in the last few years, on the use of DESs in representative solar technologies, from solar plants to last generation photovoltaics, featuring not only their passive role as green solvents, but also their active behavior arising from their peculiar chemical nature. This collection highlights the increasing and valuable role played by DESs in solar technologies, in the fulfillment of green chemistry requirements and for performance enhancement, in particular in terms of long-term temporal stability.

## 1. Introduction

The increasing awareness of global climate change, due to continuous CO_2_ emissions coming from non-renewable fuels combustion, makes the search for novel and different eco-friendly approaches for energy production impelling. Luckily, in the last decades many different devices have been developed which aim to satisfy different needs; for example, numerous photovoltaic (PV) technologies are now under investigation and many of them are already in production, even if the market is dominated by silicon-based solar panels. PV devices allow for the production of electricity with a good efficiency (the average module efficiency of Si-crystalline modules increased from 14.7% in 2010 to 20% in 2020 [1]), but electricity is difficult to store. Renewable electricity can be used for hydrogen production in water-electrolysers, representing the only clean way currently leading to green hydrogen [2]. However, this kind of water splitting is a two-step process, that in principle could lead to more energy losses than a direct process for solar-driven water splitting. Therefore, solar technologies have differentiated, focusing on the direct production of solar fuels, such as hydrogen from water splitting or CO_2_ reduction products, but also, more recently, on nitrogen fixation for ammonia production [3,4,5,6]. The development of these applications, however, requires a strong development of materials to increase the performance of devices in order to make them economically and environmentally sustainable. Since the direct conversion of energy from the sun into electricity is the most mature technology at the present time, scientific research has focused on the study of innovative materials to optimize and make these processes more sustainable. Among the solar energy conversion technologies, Dye-Sensitized Solar Cells (DSSCs) are the devices that offer the greatest possibility of intervention to improve their properties, both in terms of environmental sustainability and in terms of performance in the conversion of energy. In the last decade, perovskite solar cells (PSCs) have emerged as novel PV devices with promise of high efficiency, comparable to Si-crystalline cells, at low manufacturing costs [7,8,9,10,11,12].

Making a technology more sustainable, however, does not simply imply making it more efficient; however, especially in this phase of ecological transition, it also implies reducing the use of pollutants in the production processes of current devices, which is one of the fundamental requirements towards a sustainable development of our society. In recent decades, the breakthrough of green and sustainable chemistry in our society strongly impacted and influenced the development of the chemical industry. Volatile organic compounds (VOCs) still massively contribute to the waste stream (over 80%) in production activities despite their toxicity, high flammability, and tendency they have to accumulate in the atmosphere [13,14,15]. Accordingly, the choice of green and biodegradable solvents able to reduce the production of pollutants and, at the same time, to increase performances in synthetic and in solar energy conversion processes is crucial for the development of a sustainable future. Although water is ideally the most sustainable solvent, many synthetic and energy conversion processes are not compatible with it. Ionic Liquids (ILs) represent valuable alternatives as they exhibit interesting properties including high melting temperatures, good thermal stability, and negligible vapor pressure, thereby making them good candidates for processes under consideration [16]. Unfortunately, ILs are highly viscous, costly, and in some cases difficult to synthesize.

**Figure 1:**
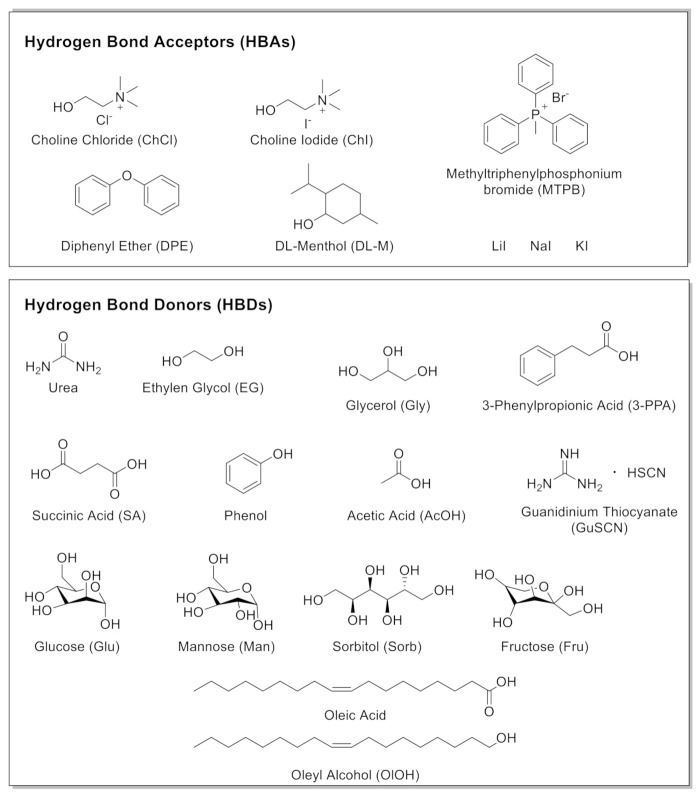
Hydrogen Bond Acceptors (HBAs) and Hydrogen Bond Donors (HBDs) used in the preparation of DESs described in this review and, when available, the corresponding name abbreviations.

Since the beginning of the new millennium, a new class of versatile and non-toxic solvents has been introduced: Deep Eutectic Solvents (DESs) [17,18,19,20]. DESs, also defined “the organic reaction medium of the century”, are a new generation of environmentally responsible neoteric fluids [21]. They are usually constructed by Lewis or Brønsted acids and bases, which can contain a variety of anionic and/or cationic species characterized by a strong hydrogen bond network. A DES is a binary or ternary combination of safe and inexpensive components comprising at least one hydrogen bond donor (HBD) and one hydrogen bond acceptor (HBA), mixed in a proper molar ratio to form a eutectic mixture with a melting point far below than that of either of the individual components. Representative hydrophilic and hydrophobic HBDs and HBAs used for the preparation of DESs described in this review are collected in Figure 1.

As detailed by Coutinho et al. [22], not all eutectic mixtures can be considered a priori DESs from a thermodynamic point of view. Indeed, the use of the adjective ’deep’ should be strictly justified only for those mixtures with a eutectic point temperature far below that of an ideal liquid mixture. These interactions decrease the lattice energy, thereby explaining the drop in melting point compared with that of their starting components [18]. DESs exhibit several attractive properties such as negligible vapour pressures, nonflammability, high thermal stability, conductivity, and easy recycling. They also show great solubility for various substances and tuneable physicochemical properties because of the possible inclusion of either hydrophilic or hydrophobic components in their structure. Even their typical high viscosity can be finely tuned by varying the DES components, the water content, or the temperature. The addition of a new different component, such as water, a carboxylic acid, or an inorganic/organic halide, to form ternary mixtures, as well as a moderate increase in temperature, often has an important and positive impact on the viscosity and density of the whole system [23].

All of these exceptional features, combined with the low cost of components, easiness of preparation with high purity, high biodegradability and low toxicity, have made DESs attractive and promising as a new generation of nonaqueous solvents/cosolvents for a broad range of applications in organic synthesis [24,25,26], main group chemistry [27,28,29,30,31,32,33,34,35], metal-, bio-, and organo-catalysis [36,37,38,39,40,41,42,43,44,45,46,47,48,49,50,51,52,53], dissolution and extraction processes [54,55,56,57,58], electrochemistry [59,60], material chemistry [61,62], photosynthesis [63], and even protein crystallization [64].

Focusing on solar technologies, in the last decade, DESs have been successfully exploited for the synthesis of active materials in thin film inorganic solar cells, such as active layers of CGS (CuGaSe_2_) [65,66], CIS (CuInSe_2_) [67], CIGS (Cu(In,Ga)Se_2_) [68,69,70,71], and CZTS (Cu_2_ZnSnS_4_) [72]. A perusal of the recent literature has shown that DESs have also been successfully employed in the synthesis of active materials for water splitting, in particular to be applied in photocatalytic [73] or photoelectrochemical [74,75,76,77,78,79,80,81] setup, or both [82,83]. Finally, active materials for CO_2_ reduction [84] and nitrogen fixation [85,86] have also been prepared via DES-based synthetic processes.

This review showcases in two Sections studies where DESs have been proven to play an active role in the final solar devices, thus excluding their use as a synthesis medium only, to afford the device materials. DESs have been successfully applied for this scope in solar devices either when acting as heat transfer fluids in concentrated solar power (CSP) plants, or when used in DSSCs, where DESs are the solvents for the electrolyte solution. To the best of our knowledge, no reports have been so far published on DES-based components in PSCs.

## 2. DES in Solar Energy Technologies

### 2.1. DES in Concentrated Solar Power (CSP) Plants

Concentrating solar power (CSP) is a well-established technology to capture solar thermal energy for use in power-producing heat processes [87,88]. The stored thermal energy can be used to power a turbine or a heat engine to generate electricity. The advantage of CSP is that the energy stored in daylight can be used during night when the thermal fluid can potentially reuse the solar energy. The main drawback is the elevated cost as compared to conventional energy sources. A possible approach to achieve a cost-effective plant is an improved efficiency of the heat transfer processes that occur in this application. In CSP, solar energy is usually concentrated using mirrors and lenses and stored in a thermal fluid. The working fluid used in the CSP plant plays an important role in determining the overall efficiency of the system. The conventional thermal fluids have low-to-moderate thermal stability and heat storage capacity, which results in high operating costs. Many studies have focused the attention on ILs as one of the alternatives for heat transfer fluids for future generations. A cheaper alternative is represented by molten salts (KNO_3_, NaNO_2_, and NaNO_3_) as heat transfer fluids in CSP. However, they suffer from a high melting point when the temperature drops below 473.15 K, leading to maintenance problems. A different approach is the use of DESs, that are inexpensive and are nowadays an effective substitute for thermal fluids. The extended benefits of using DESs would be surpassing the inherent shortcomings of comparable green solvents, particularly with respect to density and viscosity, which are essential parameters for heat transfer. In this regard, DESs have several excellent physical and chemical properties (vide supra).

For these reasons, P. Dehury et al. have published several works investigating different DESs as potential heat transfer fluids in the last three years [89,90,91,92]. In 2018, they started from the study of a dl-menthol/oleic acid 1:1 DES and, in order to improve the performance, also added different concentrations of nano-Al_2_O_3_ (70-nm diameter) to obtain 4 different nanoparticle-dispersed DESs (NDDESs) (0.001, 0.005, 0.0075, 0.01 volume fractions) [89]. These NDDESs belong to the category of nanofluids, that in the past decade have gained attention for their thermal property enhancement with respect to base fluids. In particular, they improve the convective heat transfer at the entrance region [93]. Both DESs and NDDESs were tested in their density, viscosity, and thermal conductivity. The thermal conductivity of NDDESs gave an approximate average increase of ∼10% with respect to pure DES. The specific heat capacity of DESs and NDDESs was found to increase with temperature, that is, >0.200 and >0.230 W m^−1^ K^−1^, respectively. The specific heat capacity of NDDESs was found to be higher than that of DESs, with an increase of 6%, 15%, 27%, and 50% corresponding to nanoparticle volume fractions of 0.001, 0.005, 0.0075, and 0.01, respectively. This can be attributed to the formation of an internal structure within the nanofluids, generating a specific contact between DESs and Al_2_O_3_ nanoparticles. One possible outcome is the formation of a chain-like nanostructure that resembles an infiltrating network as observed in aggregated suspensions such as nanofluids. Here, DES initiates such nanostructure as a consequence of the interaction by its menthol moiety. The nanostructure formation is much larger than that of conventional nanofluids. This implies that DES is primarily responsible for the enhanced specific heat capacity of DES-based nanofluids, and an increased specific heat capacity indicates an efficient heat transfer fluid in terms of energy storage. To complete the work, both DESs and the NDDESs with a 0.005 volume fraction of Al_2_O_3_ (resulted as an optimum choice for the thermophysical properties, flow regime, and agglomeration behavior) underwent forced convection studies, that revealed that the use of NDDESs significantly improved the convective heat transfer, particularly at the entrance region.

**Figure 2:**
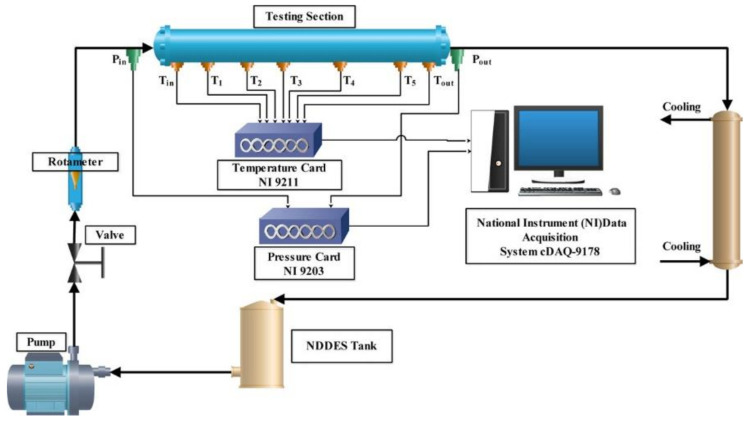
Schematic diagram of the forced convection setup. Reprinted with permission from Dehury, P.; Chaudhary, R.K.; Banerjee, T.; Dalal, A. Evaluation of Thermophysical Properties of Menthol-Based Deep Eutectic Solvent as a Thermal Fluid: Forced Convection and Numerical Studies. *Ind. Eng. Chem. Res.* **2019**, *58*, 20125–20133, doi:10.1021/acs.iecr.9b01836. Copyright 2019 American Chemical Society. Ref. [91].

In a subsequent work in 2019, Dehury studied a similar DES, a dl-menthol/oleyl alcohol 1:1 mixture [91]. In this case, no nanoparticles were added, and the pure DES was studied, comparing it with the commercially available thermal fluid Therminol VP1 (a combination of diphenyl ether and biphenyl) and the previously published data of the IL *N*-butyl-*N*,*N*,*N*-trimetylammoniumbis(trifluormethylsulfonyl)imide ([N_4111_][TF_2_N]) [94]. The physical properties of the DES were studied, in particular density, viscosity, and thermal behavior. As in the previous case, density was found to decrease with the temperature increase, and it was lower than the two reference fluids used for comparison. A lower density would eventually make the flow behavior inside the pipes easier, that is an important factor as the DES has to be pumped to the CSP to absorb the heat energy. The viscosity of DES was found to be lower compared to that of IL, while at higher temperatures DES had a viscosity similar to that of Therminol VP1. A lower viscosity reduces the pumping cost of the DES when used as a thermal fluid in CSP. Also, the study of thermal conductivity resulted in a better heat transfer for the DES (∼0.161 W m^−1^ K^−1^) than the commercially available thermal fluid Therminol VP1 and the reported IL, confirming it as a valuable alternative to current heat transfer fluids. Finally, the performance of DES has been evaluated in a forced convective heat-transfer experiment (the setup is depicted in Figure 2), showing that the thermal entrance length of the DES was very large because of its high viscosity and low thermal conductivity.

Dehury and co-workers then moved to the study of a different DES, constituted by methyltriphenylphosphonium bromide (MTPB) and ethylene glycol (EG) 1:4, and as in the first study, they also added 1% *w*/*w* Al_2_O_3_ nanoparticles (70-nm diameter) [90]. As previously reported, density of DES and NDDES decreases with temperature increase, and NDDES had a lower density compared to that of its base fluid. Unfortunately, density was 10% higher than commercial solvents. Viscosity followed the same trend, with NDDES having a lower viscosity than DES, both decreasing with temperature, but however higher than the commercial standards. The thermal properties were then analysed, revealing that both the NDDES and DES had a thermal conductivity almost 70% higher than that of the commercial fluids (∼0.185 and ∼0.200 vs. ∼0.135 W m^−1^ K^−1^), and also the heat capacity of the DES and NDDES was almost 3 times larger than that reported for commercial systems. Thus, high thermal conductivity and heat capacity values can offset the denser and more viscous NDDES as potential heat transfer fluid. Finally, a steam generation system using MTPB/EG DES as a pseudo-component was simulated, resulting in a steam generation rate increase with both the temperature and the flow rate of DES (the best value was 1.7 kg/h of steam produced at 180 °C with a corresponding DES flow rate of 1 m^3^/h), implying that the DES can be used for enhancing the heat transfer coefficient in CSP plants.

**Figure 3:**
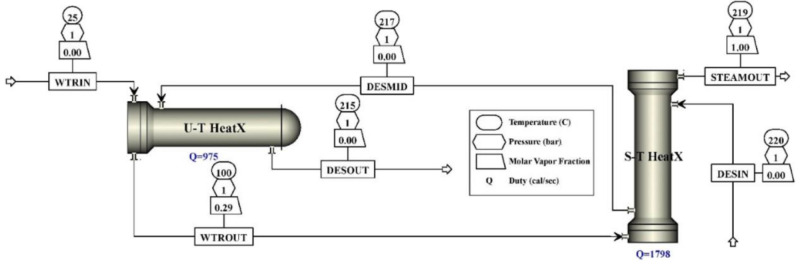
Process flow sheet for steam generation simulation. Reprinted with permission from Dehury, P.; Mahanta, U.; Banerjee, T. Comprehensive Assessment on the Use of Boron Nitride-Based Nanofluids Comprising Eutectic Mixtures of Diphenyl Ether and Menthol for Enhanced Thermal Media. *ACS Sustainable Chem. Eng.* **2020**, *8*, 14595–14604, doi:10.1021/acssuschemeng.0c05648. Copyright 2020 American Chemical Society. Ref. [92].

In their latest publication, Dehury completed the study of the previously described dl-menthol/oleyl alcohol 1:1 DES (dl-M/OlOH) by adding 0.02%, 0.05% and 0.1% *w*/*w* hexagonal boron nitride (h-BN) nanoparticles (80-nm diameter) and compared it with a novel diphenyl ether + dl-menthol 1:1.2 DES (DPE/dl-M), pure or with h-BN nanoparticles [92]. As usual, the thermophysical properties study started from density evaluation. The density of all of the solvents was found to decrease linearly with temperature increase, with each NDDES a little denser than the corresponding pure DES. As for the viscosity, the new DPE/dl-M DES proved to be less viscous than dl-M/OlOH, an indication that the presence of OlOH increased the viscosity of the final DES. The addition of nanoparticles slightly increased the viscosity for both DESs. DPE/dl-M derived NDDESs showed a significantly lower viscosity than the nanofluids based on dl-M/OlOH. Furthermore, all of the studied fluids were less viscous than commonly used ILs such as ([BMIM][Tf_2_N] and [BMIM][BF_4_]). Thermal conductivity decreased when temperature increased. All NDDESs based on dl-M/OlOH DES showed a thermal conductivity higher than the pure solvent, while the situation was more complex for NDDESs based on DPE/dl-M. NDDES with 0.02% *w*/*w* h-BN had the highest thermal conductivity, even larger than its parent base fluid except at temperatures >335 K, where both the fluids have nearly equal thermal conductivities. However, NDDESs with a higher number of h-BN nanoparticles have both positive and negative deviations from the base-fluid thermal conductivity data. This may occur due to the agglomeration of nanoparticles. Hence, these concentrations have not been studied further. In contrast to thermal conductivity, the specific heat capacity of all the investigated fluids tended to increase with the temperature. In particular, DPE/dl-M DES had the highest heat storage capacity, even a little higher than its corresponding 0.02% *w*/*w* h-BN NDDES. These last two fluids were used for a forced convection thermal study in both laminar and turbulent regime. NDDES resulted in the highest heat transfer coefficient. This fluid was finally used for a steam generation simulation (Figure 3), that interestingly revealed that, at a temperature of 494.15 K, steam generation should be equal to the amount of water input, 15 kg/h, with a 100% efficiency.

### 2.2. DES in Dye-Sensitized Solar Cells (DSSC)

In the enormous field of PV technologies, DSSCs have attracted much attention since their first appearance in the literature in 1991 [95]. These cells are composed by a photoanode, typically a thin layer of TiO_2_ sensitized by a proper dye-sensitizer in the presence of a passive cathode with Pt nanoparticles and a liquid electrolyte solution containing a redox couple that allows dye regeneration and closes the electrical circuit. The first investigated dyes were Ru-based complexes, such as the benchmark N719, that offered good performance and power conversion efficiencies (PCE) of ca. 10%, even if they have a low molar absorption coefficient. A wide applicability of these dyes was limited by the presence of the metal centre, which increases the cost of the dye and limits the tunability of their absorption properties, mainly dictated by the metal properties. A variety of metal-free organic molecules as sensitizers, with a very flexible design, were thus designed, synthesized, and tested in solar devices, exploiting the possibility to have tailor-made absorption spectra with high molar extinction coefficients as well as finely tuned other relevant properties (for example, hydrophilicity or hydrophobicity of the dye [96]). Much work has been devoted to dye optimization in order to reach higher PCEs. The present PCE record, published in 2015, is 14.7% [97]. In addition to the dye, a co-adsorbent is often present on TiO_2_ surface, typically chenodeoxycholic acid (CDCA). This molecule prevents dye aggregation, that would lower cell performance through a detrimental charge recombination [98]. One of the major drawbacks of DSSCs is that they make use of VOCs as solvents for the electrolyte solution, such as acetonitrile or mixture of nitriles. The presence of flammable, volatile and, sometimes, also toxic solvents represent a challenge for large-scale production and commercialization of DSSCs, hindering their wide application and dramatically limiting their environmental sustainability as a renewable energy source. The electrolyte solution is a key component of a DSSC. After the dye goes into its excited state upon light absorption, an electron is donated from its LUMO to the conduction band (CB) of the TiO_2_ and a hole is donated to the redox couple to restore the dye to its starting state, thus letting the device work and be ready for a new cycle. The most used redox couple is I^−^/I_3_^−^. In more recent years, other redox mediators have been successfully used, such as Co^2+^/Co^3+^ [99,100] or Cu^+^/Cu^2+^ [101], aiming to improve the maximum attainable voltage of the cell and thus the PCE. Much work has also been devoted to developing water-based electrolyte solutions in order to avoid VOCs [102,103,104]. Unfortunately, DSSC are not stable in water since the latter hydrolyses the dye-semiconductor bond leading to dye desorption and eventually to device shut off.

The first example of application of DES in DSSCs as green electrolyte solvents dates back to 2009 [105]. Since then, different papers from different groups have been published investigating a significant number of DESs. In a DSSC liquid electrolyte, the DES may act as the pure solvent or be present as a co-solvent in combination with a traditional VOC or IL. In the following section, we will review DES-based DSSC studies according to these two categories.

A brief introduction on the main parameters of a PV device can be useful for readers who are unfamiliar with this specific research field. The main characterization technique for a solar cell is the measure of a *J/V* curve, that consists in recording the current density (*J*) produced by the cell when varying the applied potential (*V*) under illumination. The illumination source is usually a solar simulator, that is a lamp with a proper filter matching the solar spectrum (AM 1.5G), whose output power is calibrated to 1000 W m^−2^, corresponding to 1 Sun. The *J/V* curve measured under illumination allows to calculate all the characteristic parameters of a PV device, namely the short circuit current density (*J_sc_*), the open circuit voltage (*V_oc_*), the fill factor (*FF*), and the PCE. The *J_sc_* is the maximum current produced by the cells, that is measured when the voltage is zero. Instead, the *V_oc_* is the maximum attainable voltage, recorded when the current is zero. The *FF* is the ratio between the output power (*P_out_*) of the cell and the product *J_sc_* × *V_oc_*. Finally, the efficiency PCE is defined as the ratio between the output and input power densities, *P_out_* and *P_in_*, respectively. Since the output power density is equal to *J_sc_* × *V_oc_* × *FF*, PCE is given by the following relationship (1).

(1) $$\mathrm{PCE}=\frac{J_{sc}V_{oc}FF}{P_{in}}$$

The external quantum efficiency of a DSSC, that is the efficiency from incident photons to generated current, is also a relevant device parameter. In the DSSC literature, this efficiency is referred to as Incident Photon-to-Current Conversion Efficiency (IPCE), and is a function of the wavelength, λ. In this measure, the photocurrent is recorded in open circuit conditions while the cell is illuminated by monochromatic light. IPCE is then calculated point by point as the ratio between the number of collected electrons and the number of photons at a given λ.

#### 2.2.1. DSSCs Using DES as an Electrolyte Solvent

**Figure 4:**
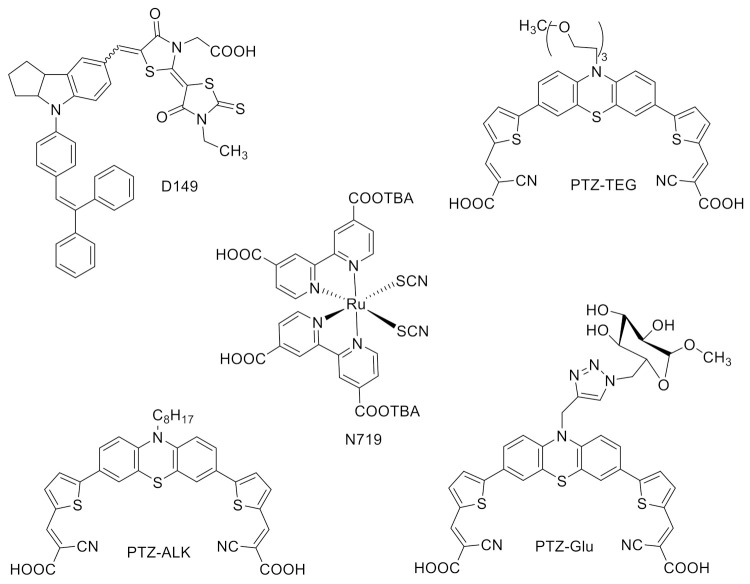
Structures of typical dyes used in DES-based DSSCs.

DESs have been successfully used as solvents in DSSC electrolytes in a significant number of studies. The sensitizers used for these DSSCs are depicted in Figure 4, while Table 1 summarizes the main results obtained in the reviewed papers.

In their pioneering work on DESs as electrolyte solvent, H.-R. Jhong et al. tested a novel DES containing ChI/Gly in 1:3 ratio, diluted with 15% *w*/*w* water to keep it liquid at room temperature [105]. In fact, this DES represented a minority part of the electrolyte solution volume, mainly constituted by the IL 1-methyl-3-propylimidazolium iodide (PMII) used as an iodide source (13:7 *v*/*v*). The metal-free indoline dye D149 (Figure 4) was chosen as a sensitizer because of its very high molar extinction coefficient (68,700 M^−1^ cm^−1^ at 526 nm), that allows for the use of thin layers of TiO_2_, more suitable in case of highly viscous electrolyte solutions. A total of 2 different electrolyte compositions were used: (i) an acetonitrile-based electrolyte containing 0.1 M LiI, 0.6 M PMII, 0.05 M I_2_, 0.05 M 4-*tert*-butylpyridine (4-tBP) in a mixture of acetonitrile and valeronitrile (*v*/*v*, 85:15); (ii) the DES-based electrolyte containing 0.2 M I_2_, 0.5 M *N*-methylbenzimidazole (NMB) in a mixture of PMII and ChI/Gly (*v*/*v*, 13:7). The DES-based DSSC showed an efficiency of 3.9%, while the control cell with acetonitrile yielded an efficiency of 4.9%. The IPCE value of DES-based DSSC reached a remarkable value of 73% at 550 nm. The main difference between DES and acetonitrile-based IPCE spectra was a drop in IPCE of the former electrolyte from 400 to 450 nm, that could be due to the light absorption of the I_3_^-^ in IL, present in higher concentrations in the solution. A deeper investigation was dedicated to the study of viscosity and conductivity of the two electrolyte solutions. As expected, DES-based solution was more viscous than that based on VOC. The conductivity of the ChI/Gly-based electrolyte was up to 5.30 mS cm^−1^, to be compared with a value of 25.1 mS cm^−1^ for the acetonitrile-based electrolyte. The apparent diffusion coefficient of I_3_^-^ in the DES-based electrolyte was higher than in other IL-based electrolyte solutions (1-ethyl-3-methylimidazolium tetracyanoborate (EMIB(CN)_4_)/PMII).

Following this example, our group has, in recent years, tested different types of DESs, hydrophilic [106], hydrophobic [107] and the so-called natural DESs (NADESs) [108]. The first studies focused on the optimization of the device parameters in order to optimize the performance in the novel unconventional electrolyte medium. As a representative DES we selected the commonly used ChCl/Gly 1:2 mixture, which was diluted with 40% *w*/*w* water to lower the viscosity, thereby making the solution suitable to smoothly fill the cell with the liquid electrolyte. The conventional DSSC dye, typically carrying a terminal hydrophobic alkyl moiety to depress recombination pathways from the semiconductor to the electrolyte [112,113], was replaced by a hydrophilic counterpart, in order to have a better interface interaction with the new aqueous DES medium. In particular, we have used the dye PTZ-TEG, a push-pull organic molecule with a phenothiazine donor part functionalized by a hydrophilic glycol chain (Figure 4). The study started by comparing the performance of DSSCs with two different co-adsorbents, the conventional CDCA and glucuronic acid (GlcA). We introduced GlcA as a hydrophilic co-adsorbent able to avoid, in a polar aqueous-DES medium, dye aggregation, thus playing the same role conventionally covered by CDCA with alkyl-functionalized sensitizers. In fact, PCE was found to be the same with both co-adsorbents, CDCA and GlcA, the latter, however, leading to a lower dye loading, thus indicating that it actually promoted a more effective supramolecular dye organization on the TiO_2_ surface. The conventional redox pair I^−^/I_3_^−^ was selected as an electrolyte redox pair, and as an iodide source we tested both an inorganic (KI) and organic (imidazolium iodide) common salts, with the best efficiency (1.0%) given by PMII 2 M (iodide concentration was 0.02 M for all the experiments). The addition of guanidinium thiocyanate (GuSCN), a commonly used additive in DSSCs, led to a small PCE improvement (1.3%). To increase the *V_oc_*, also pyridine derivatives (4-tBP, 4-picoline, pyridine) were added in the electrolyte solutions. Upon coordination to the TiO_2_ surface through the ring nitrogen lone pair, the energy of the CB and Fermi level of the semiconductor is shifted to higher values. This results in an increase in the *V_oc_*, which is proportional to the difference between the TiO_2_ CB and the Nernst level of the redox couple [114]. Pyridine derivatives did effectively increase the *V_oc_*, but they also decreased the *J_sc_*, most likely because of dye desorption from the TiO_2_ surface due to the hydrolysis in basic aqueous media of the bond between the dye and TiO_2_ [115]. A detailed optimization of the semiconductor thickness and morphology (addition of a scattering layer and of a compact blocking layer) allowed to reach an optimized efficiency of 1.7%. The IPCE measurement of the best cell gave IPCE values >40% in the range 480–550 nm.

Maintaining the same optimized device parameters, we then turned our attention to a less conventional hydrophobic DES, dl-menthol/AcOH 1:1, diluted with 10% *v*/*v* ethanol to have proper viscosity and solubilizing properties for the electrolyte solutes [107]. The advantage of using a hydrophobic DES is the possibility of employing widely available hydrophobic DSSC sensitizers, that is those typically studied in combination with VOC-based electrolytes, thus including the most performing reported systems. For a more fruitful comparison, we chose a sensitizer similar to that hereabove described, where the phenothiazine donor moiety carried a terminal C_8_-alkyl chain (PTZ-ALK) (Figure 4). The performance of the DES-based cell was compared with an analogous cell with the same electrolyte composition but using the standard acetonitrile/valeronitrile 85:15 mixture as a solvent. The electrolyte solution was composed by 1.0 M 1,3-dimethylimidazolium iodide (DMII), 0.03 M I_2_, 0.1 M GuSCN, and 0.5 M 4-tBP. As expected, the efficiency of the VOC-cell resulted higher than that with DES (4 vs. 2.5%), the latter having almost half the photocurrent, likely due to the higher viscosity of the medium. Interestingly, the DES-based cell exhibited a higher photovoltage. A further analysis with electrochemical impedance spectroscopy (EIS) showed that the charge recombination resistance between the sensitized oxide and the electrolyte, which controls the extent of the detrimental charge recombination, was more than twice larger in cells with DES than with the conventional VOC medium. This confirmed that charge recombination rates were much smaller in the former cell, accordingly with the higher *V_oc_* recorded.

**Figure 5:**
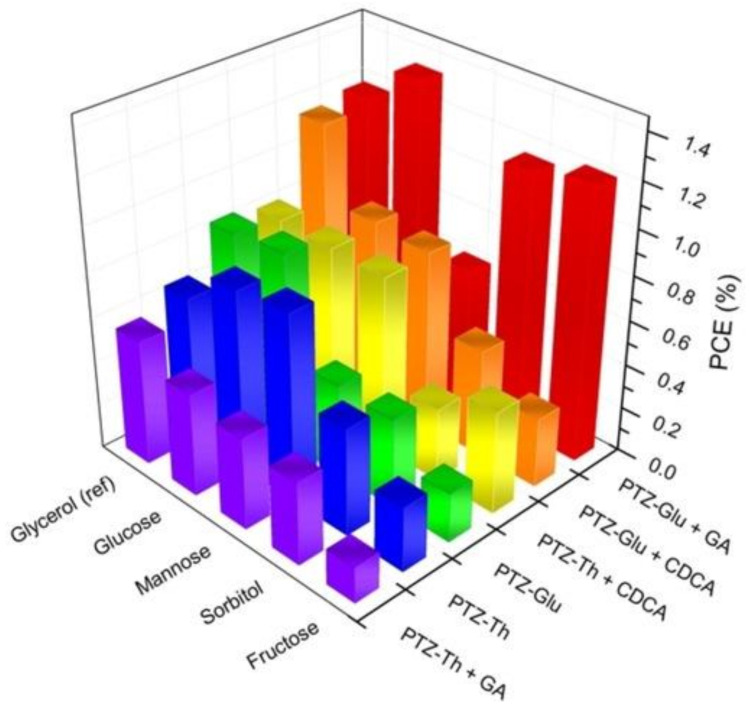
Histograms comparing the efficiency as a function of dye, co-adsorbent and carbohydrate-based NADES. Reprinted with permission from ref. [108].

In a subsequent investigation, we tested a few NADESs based on ChCl in combination with different conventional carbohydrates [108]. In particular, after selecting ChCl/Gly 1:2 + 40% water as a reference medium, we studied the following NADESs: ChCl/Glu 1:2 + 30% water, ChCl/Sorb 1:1 + 30% water, ChCl/Fru 2:1 + 20% water, and ChCl/Man 2:5 + 20% water. As in our previous work [106], the sensitizer was designed so as to optimize the interface interaction between the different components of the cell in order to increase charge transfer pathways. A phenothiazine push-pull dye bearing a sugar moiety on the central ring (PTZ-Glu) was used and compared with the more conventional alkyl-functionalized PTZ-ALK (Figure 4). The same approach was used for co-adsorbents, comparing GlcA and CDCA. These findings in the presence of different combinations of the strategic device components (dye, co-adsorbent, and NADES) are summarized in Figure 5. We concluded that a collective interplay involving the sugar-based sensitizer, co-adsorbent and NADES is responsible for a significantly enhanced device performance. IPCE plots were recorded for the best performing PTZ-Glu + GlcA cells showing a wide absorption throughout the entire visible range, and a maximum at ca. 530 nm. The highest IPCE values were recorded for the cell using ChCl/Glu NADES, with a peak of ca. 30%.

**Figure 6:**
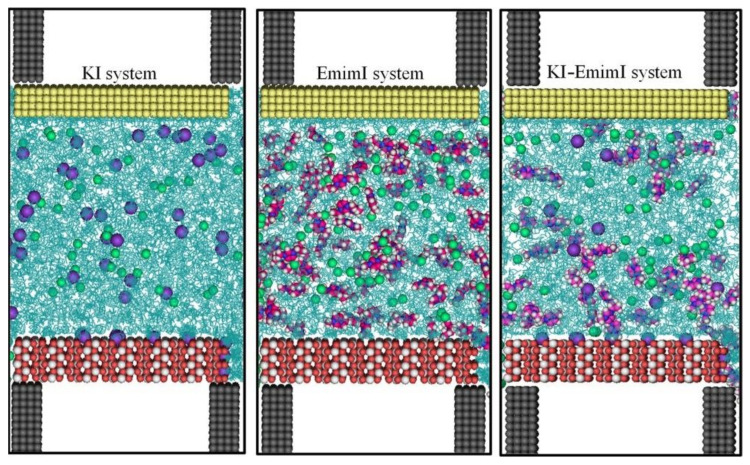
Snapshots of the electrolyte mixtures confined inside TiO_2_ and Pt walls after 20 ns. Pt, Ti, O, I, K, N, C, and H atoms are depicted in yellow, white, red, green, purple, blue, pink, and white spheres, respectively. The DES is shown as cyan sticks. Reprinted with permission from Heydari Dokoohaki, M.; Mohammadpour, F.; Zolghadr, A.R. Dye-Sensitized Solar Cells Based on Deep Eutectic Solvent Electrolytes: Insights from Experiment and Simulation. *J. Phys. Chem. C* **2021**, *125*, 15155–15165, doi:10.1021/acs.jpcc.1c02704. Copyright 2021 American Chemical Society. Ref. [109].

The interest in DES-based DSSCs has very recently increased, with a number of reports being published in 2021. M. Heydari Dokoohaki and co-workers studied the effect of an inorganic and an organic iodide source (namely KI and 1-ethyl-3-methylimidazolium iodide, EmimI) in a ChCl/EG 1:2 DES, both with an experimental and an atomistic molecular dynamics approach [109]. The sensitizer was the commercial N719 (Figure 4) and the electrolyte solutions were composed by (i) 0.6 M KI, 0.02 M I_2_; (ii) 0.6 M EmimI, 0.02 M I_2_; (iii) 0.3 M KI, 0.3 M EmimI, 0.02 M I_2_. ChCl/EG DES was diluted with 5% *v*/*v* water in order to modify the solubility of the electrolyte components. *J/V* curves showed that the DES electrolyte with KI as an iodide source showed better PV performance than the DES electrolyte containing EmimI thanks to an almost twice as larger *J_sc_*. This could be explained considering that the adsorption of smaller cations, such as K^+^, on the TiO_2_ surface leads to a shift of the CB level of the semiconductor, thus facilitating the electron injection from the LUMO of the dye into the CB of TiO_2_. Since the observed performance could also come from different physicochemical properties of the investigated electrolyte solutions, viscosity and conductivity were measured. The results showed that the increase in the size of cation resulted in a concomitant reduction in the viscosity of the corresponding electrolyte, and in a higher conductivity. However, a comparison of the measured efficiency for DES electrolytes demonstrated a contrast between the higher fluidity or conductivity of the electrolytes and DSSC performance. An experimental explanation of the PV properties came from EIS measurement. EmimI induced a higher charge-transfer resistance at the platinum electrode that can be explained as a deterioration of the catalytic performance at the counter electrode, with a subsequent decline in DSSC performances. Furthermore, the KI-based cell had both a higher electron lifetime and a total electron density that was 1.7 times greater than that of the EmimI system. Altogether, these characteristics explained why KI-based cells had a higher photocurrent when compared to EmimI-based devices. However, the authors wanted to conduct a deeper investigation on the phenomena occurring at the interfaces of the cell by using Molecular Dynamics simulations, whose results are depicted in Figure 6. They found that the potassium and Emim^+^ cations were mainly located at the TiO_2_ surface whereas iodide anions were concentrated at the Pt surface. An excess concentration of DES components was also found at the Pt surface. Furthermore, a significant layering of K^+^ cations was observed near the TiO_2_ electrode in contrast to Emim^+^ cations. I^−^, EG, and Cl^−^ were found to form the next layers. As a result, the smaller K^+^ cations were likely to coordinate more strongly to Cl^-^ and also EG molecules of the DES than the larger Emim^+^ cations. In this way, K^+^ ions were able to migrate less freely due to the solvation effect with respect to Emim^+^. Iodide ions could thus diffuse with lower hindrance for ion pair formations in the systems containing K^+^ in line with the calculated diffusion coefficient, accounting for a better performance.

D. J. Boogaart et al. investigated the effect of different amounts of water in 3 previously described DESs (ChCl/urea 1:2, ChCl/EG 1:2 and ChCl/ Gly 1:2) and in an unprecedented DES based on ChCl and GuSCN 1:1 [110]. Each DES was tested as itself, with the exception of ChCl/GuSCN that was solid at room temperature, or with different amounts of water (from 10 to 40% *w*/*w*). The electrolyte solution contained KI 0.5 M and I_2_ 0.025 M in DES or in water as a control and the sensitizer was the benchmark dye N719 (Figure 4). For a proper comparison of the results, also a commercial reference electrolyte solution was used (Iodolyte, Solaronix). The PV measurements showed that the highest performances were obtained for electrolytes containing >20% *w*/*w* water, likely because of a lower viscosity. With a 30% water concentration, the electrolytes based on ChCl/urea, ChCl/EG and ChCl/GuSCN produced higher device performance compared to the aqueous control. ChCl/urea DES led to a markedly improved *V_oc_*, likely arising from positive alterations of TiO_2_ electronic properties. The best performance was recorded with cells filled with ChCl/GuSCN. To better understand the potential chemical effects of the DES constituents, aqueous electrolyte solutions based on every single component were prepared using the same concentration of that specific molecule used in 70% *w*/*w* DES solutions. The results indicated that none of the individual components provided any real benefit to PV performance, and many were, in fact, detrimental. For example, for the aqueous solution corresponding to ChCl/urea DES, ChCl essentially had no effect on photovoltage, while urea provided a small increase. Furthermore, ChCl and GuSCN produced the two lowest performances, showing detrimental effects as individual components. However, when used in combination as ChCl/GuSCN DES, a massive enhancement in PV performance was observed, clearly indicating the presence of synergistic effects. *J/V* measurements in 100% *w*/*w* DES cells highlighted that two aspects of limited diffusion were occurring: (i) hindered charge-carrier transport evidenced by the hump in *J*/*V* values and (ii) poor penetration of the viscous electrolytes into TiO_2_ mesopores.

In 2021, H. Cruz et al. published a novel approach for DES application in DSSC, using alkali iodide DESs as alternative electrolytes in place of more commonly used DESs containing other halides (such as chloride), thus eliminating the addition of any external iodide source [111]. The investigated DESs in N719-sensitized cells were LiI/EG 1:3, LiI/EG 1:10, NaI/EG 1:3, and KI/EG 1:5. The electrolyte solutions contained different amounts of I_2_ (0.1–10% mol). A VOC-based reference electrolyte (0.8 M LiI, 0.05 M I_2_) in an acetonitrile/valeronitrile (85:15, % *v*/*v*) mixture was studied. DES-based DSSCs had a higher *V_oc_* than VOC-based reference cells. In particular, the photovoltage increased with the cation size, from Li^+^ to K^+^. This was ascribed to the fact that the smaller Li^+^ is more easily adsorbed onto the semiconductor surface, and this led to a beneficial shift of the TiO_2_ flat band potential, thus decreasing the voltage. The authors also investigated the effect of different iodine concentration, envisaging better performances by increasing the iodine concentration. In fact, in KI/EG 1:5 cells, the conversion efficiency increased on going from 0.1 to 0.5% mol I_2_ but, upon further increasing the concentration, the performances dramatically dropped, with eventually a PCE of 0% at 10% mol I_2_. This was rationalized in terms of a larger electron recombination, due to an increase in triiodide concentration near the semiconductor surface, and to less transparent and heavily coloured electrolyte solutions thus excessively filtering illumination. Interestingly, DSSCs based on KI/EG 1:5 + 0.5% mol I_2_ showed a marked long-term temporal stability, with still residual 50% efficiency after one month and 40% after seven months, in particular, when compared to reference VOC-cells, whose efficiency dropped to zero after one month because of solvent evaporation.

#### 2.2.2. DSSCs Using DES as a Co-Solvent in Combination with VOCs or ILs

P. H. Tran developed a few different systems where DESs were used in combination with a standard organic solvent, namely ethanol, acetonitrile and 1-ethyl-3-methylimidazolium tetracyanoborate (EMITCB) [116,117,118]. The PV parameters of the obtained DSSCs are collected in Table 2. This study started in 2018 with the use of 3 different DESs, diluted with 50% *w*/*w* ethanol, constituted by ChCl/ 3-phenylpropionic acid (3-PPA), and succinic acid (SA) or EG in a 1:1:1 ratio [110]. The electrolyte of N719-sensitized DSSCs consisted in 0.3 M tetrabutylammonium iodide (TBAI), 0.05 M I_2_ and 0.25 M 4-tBP. The best cell, using a ChCl/EG DES, reached a PCE of 2%. This result was ascribed to the lower viscosity and the highest conductivity of the DES.

A subsequent study by Tran’s group involved a ChCl/phenol 1:2 DES in acetonitrile (up to 50% *v*/*v*) [117]. In the presence of 0.6 M TBAI, 0.1 M GuSCN, 0.5 M 4-tBP, and 0.03 M I_2_ electrolyte (I_2_ was tested up to 0.1 M in DES/acetonitrile 1:1 mixtures) cells with pure acetonitrile as a solvent offered the best performance. Indeed, the presence of any amount of DES decreased device current and voltage. The highest voltage in DES/acetonitrile cells was observed with mixtures containing higher concentrations of DES. The most interesting results came from the study of the stability of DSSCs over 1300 h (54 days) in dark. More importantly, DSSCs containing DES as a solvent component exhibited a remarkable long-term stability, with stable current values after more than 1000 h, in contrast with a much lower stability of acetonitrile devices, where values dropped significantly after the first 300 h. Moreover, cells with 20% DES showed increased efficiency with time, passing from 6.9 to 7.8% after 1300 h. In the acetonitrile cell, PCE lowered with time to such an extent that, after 1300 h, it became as high as that of 20%-DES devices. The authors postulated that the structure of DES comprising choline and phenyl groups might interact with TiO_2_ surface, thereby preventing back electron transfer to the redox mediator. This hypothesis was confirmed by DFT calculations on the binding between choline or phenyl groups with TiO_2_ and on the subsequent effect on the TiO_2_ band gap. Such co-adsorption prevented the oxidized form of the redox pair from approaching the TiO_2_ surface, thus minimizing the dark current, due to charge recombination, and also shifting the Fermi level to a lower energy.

More recently, the same group reported the application of two DESs, ChCl/urea 1:2 and ChCl/EG 1:2, in different volume ratios with the IL EMITCB (from 0 to 60%) [118]. With N719 and a standard electrolyte (1 M PMII, 0.2 M I_2_, 0.1 M GuSCN, and 0.5 M NMB), the performance of DES-based DSSCs compared well with that of cells filled only with the IL. In particular, at lower DES concentrations, energy conversion performance was only slightly worse than that of DES-free cells, whereas PCE decreased significantly upon increasing the DES content, mainly due to a photocurrent drop. This finding was explained based on the different physicochemical properties of the investigated solvents: EMITCB has a low viscosity and a high conductivity, while ChCl/EG is slightly more viscous and less conductive. The worst performing DES, ChCl/urea, is a very viscous solvent with a low conductivity. The *J_sc_* trend was also confirmed by IPCE curves. However, it should be noted that DES-DSSCs containing urea as a DES component exhibited the best photovoltage values, as also reported by Boogaart (see Section 2.2.1) [110]. EIS data analysis of the electrolyte solvent based on ChCl/urea showed that the density of oppositely charged ions at the interface was lower, thus leading to a decreased electron loss probability and, in turn, to a higher *V_oc_*. The phenomenon observed with the ChCl/urea DES is similar to the shielding effect usually observed with conventional nitrogen heterocyclic additives such as NMB or 4-tBP. Since NMB was present in all the electrolytes the authors conjectured that either urea directly acts as an additive or might simply promote the activity of NMB. Furthermore, the ChCl/urea mixture is less polar than ChCl/EG, thus providing a good insulator environment under the internal electric field of the device, offering a higher resistance to the leakage current even better than pure IL.

## 3. Conclusions

The application of DESs to several fields of science remains a rather young and exciting technology with promising potential at an industrial scale. Mixing and heating two solids to make a liquid on a large scale, however, is not as trivial as one might think. DESs are, indeed, tuneable mixtures par excellence including various Brønsted or Lewis acids and bases with different physicochemical characteristics (from hydrophilic to hydrophobic) and, sometimes, thermally sensitive components. This is why in order to drive DESs towards an industrial reality, research has been focusing in setting up scalable and different preparation methods, able to be customized according to the specific mixture needed, that so far include mechanochemical methods and also continuous flow technologies by twin screw extrusion [119].

This review showcases that DESs have proven to be highly attractive components also for solar devices, both in CSP plants and DSSCs. Although a real CSP plant containing DESs as a heat transfer fluid has not yet been realized, current studies on DESs have disclosed very promising properties for this application. The interest for DESs in DSSCs has been growing in the last years, with several DESs tested as electrolyte solvents in combination with different sensitizers and electrolyte components. DESs have been used both as a pure solvent (with or without the addition of water) or in mixture with ILs or VOCs. What appears particularly evident from the reported data is a general improvement of the long-term stability of the solar devices, even when DES represents a minor part of the electrolyte medium. The efficiencies so far reported for DES-based DSSCs, with the DES acting as the only solvent (with or without water), are not generally very high, in any case, not exceeding the PCE value of 2.5%. However, we believe that the growing interest in the field as well as the steady improvements of performances could lead in the near future to better performances. Higher PCEs have been reached using mixture of DES and other conventional solvents but, in those cases, VOC-related problems, such as flammability, toxicity, and volatility, remain active.

As DESs can work as (co)-solvents, catalysts and even reagents, in order to match the design of a certain eutectic mixture with the desired application, a deeper knowledge of DESs at the fundamental level (structure and dynamics, the intra- and intermolecular network of bonds taking place within and around DES) as well as of the role played by water in DES formation and stability (in the case of hydrophilic mixtures) cannot be disregarded. DES-based solar devices certainly represent an emerging field of highly sustainable and eco-friendly green technologies, which could contribute to the increasing role of clean and renewable energies in the scientific community and in the societal challenges of the next decades, provided that an investment on fundamental science is also made. In this regard, it is necessary to obtain a deeper knowledge of the active role of DESs, in particular on the intermolecular interactions with the different components of the solar architecture, and their beneficial effects on the key intermolecular charge transfer mechanisms. Thanks to these studies, it will be possible to design DESs with the optimal chemical and physical characteristics in order to maximize the performance of completely eco-sustainable solar devices.

## Figures and Tables

**Table 1:** Photovoltaic parameters of DSSCs containing DES as a solvent ^1^.

DES	Diluting Agent	Iodide Source	TiO_2_ Layer ^2^	Dye/ Co-Adsorbent	*J_sc_* (mA cm^−2^)	*V_oc_* (V)	*FF*	PCE (%)	Ref.
ChI/Gly 1:3	15% *w*/*w* water	PMII 13:7 *v*/*v* ^3^	6 µm T + 5 µm S	D149/ no co-adsorbent	12.0	0.533	0.58	3.88	[105]
ChCl/Gly 1:2	40% *w*/*w* water	KI 2 M ^4^	5 µm T	PTZ-TEG/ GlcA 1:10	1.9	0.429	0.64	0.5	[106]
		PMII 2 M ^4^			3.3	0.478	0.67	1.0	
		PMII 2 M ^5^			4.1	0.495	0.65	1.3	
		PMII 2 M ^4^	2.5 µm T		4.6	0.469	0.65	1.4	
		PMII 2 M ^5^			5.1	0.504	0.66	1.7	
dl-menthol /AcOH 1:1	10% *v*/*v* EtOH	DMII 1.0 M ^6^	2.5 µm T	PTZ-ALK/ CDCA 1:1	6.6	0.585	0.65	2.5	[107]
ChCl/Gly 1:2	40% *w*/*w* water	PMII 2 M ^4^	2.5 µm T	PTZ-Glu/ GlcA 1:10	4.3	0.483	0.57	1.2	[108]
ChCl/Glu 1:2	30% *w*/*w* water				4.0	0.527	0.64	1.4	
ChCl/Sorb 1:1	30% *w*/*w* water				3.6	0.502	0.64	1.2	
ChCl/Fru 2:1	20% *w*/*w* water				3.4	0.523	0.68	1.2	
ChCl/Man 2:5	20% *w*/*w* water				1.8	0.546	0.62	0.6	
ChCl/EG 1:2	5% *v*/*v* water	KI 0.6 M ^4^		N719/ no co-adsorbent	3.3	0.72	0.65	1.60	[109]
		KI 0.3 M + EmimI 0.3 M ^4^	T		2.6	0.74	0.6	1.16	
		EmimI 0.6 M ^4^			1.7	0.75	0.58	0.73	
ChCl/Urea 1:2	30% *w*/*w* water	KI 0.5 M ^7^	T	N719/ no co-adsorbent	2.8	0.680	0.65	1.24	[110]
ChCl/EG 1:2					3.8	0.576	0.69	1.51	
ChCl/Gly 1:2					2.0	0.545	0.82	0.90	
ChCl/GuSCN 1:1					4.7	0.588	0.63	1.72	
LiI/EG 1:3	no diluting agent	DES defined ^8^	10 µm T + 5 µm S	N719/ no co-adsorbent	4.0	0.457	0.60	1.12	[111]
LiI/EG 1:10		DES defined ^9^			4.5	0.572	0.62	1.61	
NaI/EG 1:3		DES defined ^8^			4.0	0.460	0.62	1.16	
KI/EG 1:5		DES defined ^9^			6.0	0.545	0.69	2.30	

^1^ AM 1.5G 1000 W m^−2^ simulated sunlight. In case of many different variables reported in the same paper, only the most representative results are listed in the Table; ^2^ T = transparent layer, S = scattering layer; ^3^ other electrolyte components: 0.2 M I_2_, 0.5 M NMB; ^4^ other electrolyte components: 0.02 M I_2_; ^5^ other electrolyte components: 0.02 M I_2_, GuSCN 0.1 M; ^6^ other electrolyte components: 0.03 M I_2_, 0.1 M GuSCN, 0.5 M 4-tBP; ^7^ other electrolyte components: 0.025 M I_2_; ^8^ other electrolyte components: 1 mol% I_2_; ^9^ electrolyte composition: 0.5 mol% I_2_.

**Table 2:** Photovoltaic parameters of DSSCs containing DES as a co-solvent ^1^.

DES	Co-Solvent	DES Concentration	TiO_2_ Layer ^2^	*J_sc_* (mA cm^−2^)	*V_oc_* (V)	*FF*	PCE (%)	Ref.
ChCl/3-PPA 1:1	ethanol	50% *w*/*w* ^3^	T + S	5.07	0.72	0.47	1.7	[116]
ChCl/SA 1:1				4.37	0.69	0.52	1.6	
ChCl/EG 1:1				4.53	0.76	0.57	2.0	
ChCl/phenol 1:2	acetonitrile	0% *v*/*v* ^4^	12 µm T + 4 µm S	15.93	0.79	0.65	8.23	[117]
		20% *v*/*v* ^4^		14.44	0.71	0.67	6.92	
		40% *v*/*v* ^4^		12.64	0.73	0.65	5.99	
		50% *v*/*v* ^4^		12.41	0.76	0.62	5.87	
		50% *v*/*v* ^5^		12.51	0.75	0.65	6.01	
ChCl/EG 1:2	EMITCB	0% *v*/*v* ^6^	T + S, 12 µm total	11.5	0.69	0.68	5.4	[118]
		33% *v*/*v* ^6^		11.2	0.66	0.69	5.1	
		50% *v*/*v* ^6^		10.0	0.66	0.61	4.0	
		60% *v*/*v* ^6^		8.5	0.65	0.67	3.6	
ChCl/urea 1:2		33% *v*/*v* ^6^		10.3	0.72	0.69	5.1	
		50% *v*/*v* ^6^		9.0	0.75	0.64	4.3	
		60% *v*/*v* ^6^		5.7	0.74	0.73	3.0	

^1^ AM 1.5G 1000 W m^−2^ simulated sunlight; ^2^ T = transparent layer, S = scattering layer, sensitizer: N719; ^3^ other electrolyte components: 0.3 M TBAI, 0.05 M I_2_ and 0.25 M 4-tBP; ^4^ other electrolyte components: 0.6 M TBAI, 0.1 M GuSCN, 0.5 M 4-tBP and 0.03 M I_2_; ^5^ other electrolyte components: 0.6 M TBAI, 0.1 M GuSCN, 0.5 M 4-tBP and 0.05 M I_2_; ^6^ other electrolyte components: 1 M PMII, 0.2 M I_2_, 0.1 M GuSCN, and 0.5 M NMB.

## Data Availability

Not applicable.
